# MicroRNA-Mediated Health-Promoting Effects of Phytochemicals

**DOI:** 10.3390/ijms20102535

**Published:** 2019-05-23

**Authors:** Hara Kang

**Affiliations:** Division of Life Sciences, College of Life Sciences and Bioengineering, Incheon National University, Incheon 22012, Korea; harakang@inu.ac.kr; Tel.: +82-32-835-8238; Fax: +82-32-835-0763

**Keywords:** phytochemicals, microRNA, health-promoting effects

## Abstract

Phytochemicals are known to benefit human health by modulating various cellular processes, including cell proliferation, apoptosis, and inflammation. Due to the potential use of phytochemicals as therapeutic agents against human diseases such as cancer, studies are ongoing to elucidate the molecular mechanisms by which phytochemicals affect cellular functions. It has recently been shown that phytochemicals may regulate the expression of microRNAs (miRNAs). MiRNAs are responsible for the fine-tuning of gene expression by controlling the expression of their target mRNAs in both normal and pathological cells. This review summarizes the recent findings regarding phytochemicals that modulate miRNA expression and promote human health by exerting anticancer, photoprotective, and anti-hepatosteatosis effects. Identifying miRNAs modulated by phytochemicals and understanding the regulatory mechanisms mediated by their target mRNAs will facilitate the efforts to maximize the therapeutic benefits of phytochemicals.

## 1. Introduction

Phytochemicals are plant-derived compounds abundant in a variety of fruits, vegetables, herbs, and many other plants [1]. More than 10,000 phytochemicals have been identified to date, and their medicinal properties including anti-inflammatory and anticancer effects, are being investigated [2]. Polyphenols, alkaloids, terpenoids, and organosulfur compounds are well-known phytochemical groups with anticancer properties [3]. For example, polyphenols such as resveratrol and curcumin modulate oxidative stress and inflammatory signaling, and perform antioxidant and anti-inflammatory activities [4,5]. In addition, they inhibit angiogenesis by regulating the gene expression of key regulators, such as vascular endothelial growth factor and hypoxia inducible factor 1 subunit alpha, and promote apoptosis by modulating levels of Bcl2, BCL2-associated X (Bax), and p53 [6,7]. These activities cooperatively lead to the prevention of carcinogenesis. Due to the medicinal properties of phytochemicals and their availability as therapeutic agents, their underlying molecular mechanisms are being investigated.

Emerging data suggest that some phytochemicals regulate the expression of various microRNAs (miRNAs). MiRNAs are small, noncoding RNAs involved in a wide variety of cellular processes, such as development, proliferation, differentiation, and apoptosis [8]. MiRNAs regulate gene expression by binding to target mRNAs via base pairing with the 3′-untranslated regions (3′UTRs) of the target mRNAs [9]. The miRNA-target mRNA interaction usually results in the degradation or translational repression of the target mRNA. As a single miRNA can target multiple mRNAs implicated in various cellular phenomena, miRNAs are crucial post-transcriptional regulators for the fine-tuning of normal cellular physiology [8]. Expression of miRNAs is tissue- or developmental stage-specific, and their aberrant expression is associated with the development of pathogenic conditions [10]. For example, abnormal expression of several miRNAs is associated with the initiation and progression of carcinogenesis and metastasis [11]. Therefore, miRNA profiles in a wide range of diseases have been analyzed for their potential use as diagnostic or prognostic indicators.

Extensive emerging evidence suggests that phytochemicals affect the expression profiles of miRNAs in pathological conditions, especially cancer. Moreover, several studies have identified novel target mRNAs of phytochemical-modulated miRNAs and investigated the underlying mechanism for the miRNA-mediated therapeutic activities of a few phytochemicals. Herein, we review the current information on phytochemicals that benefit human health by modulating miRNA expression. Specifically, we discuss phytochemicals that exhibit anticancer, photoprotective, and anti-hepatosteatosis effects. This not only helps to understand health-promoting effects of phytochemicals at the molecular level, but also allows us to think about further research and therapeutic development.

## 2. Phytochemicals and miRNAs

Although emerging data clearly suggest that miRNA expression is specifically regulated by phytochemicals, relatively little is known about their molecular relationships to date. Baselga-Escudero et al. suggested that polyphenols directly bind to mature miRNAs and that the chemical structure of polyphenols influences the expression of miRNAs [12]. In 1 H NMR spectroscopy studies, direct binding of resveratrol and (-)-epigallocatechin-3-gallate (EGCG) to miR-33a and miR-122 was observed [12]. While resveratrol binds miR-33a and miR-122 through an A ring interaction and increases the expression levels of these miRNAs, EGCG decreases miR-33a and miR-122 expression by direct binding through an interaction with all rings in the molecules. Additionally, phytochemicals have been shown to affect miRNA levels by regulating molecules associated with controlling miRNA expression [13,14]. For example, EGCG binds the HIF-1α protein, which is a transcriptional activator of miR-210, and interferes with the hydroxylation of Pro residues in the oxygen-dependent degradation domain [14]. As the hydroxylation of Pro residues is essential for proteasome-mediated degradation of HIF-1α [15], EGCG binding increases HIF-1α expression and consequently enhances miR-210 expression levels. Pan el al. demonstrated that resveratrol regulates the expression of c-Myc, which is a transcriptional activator of miR-17, resulting in the suppression of oncogenic miR-17 levels [13].

## 3. Phytochemicals with Anticancer Effects

**Figure 1:**
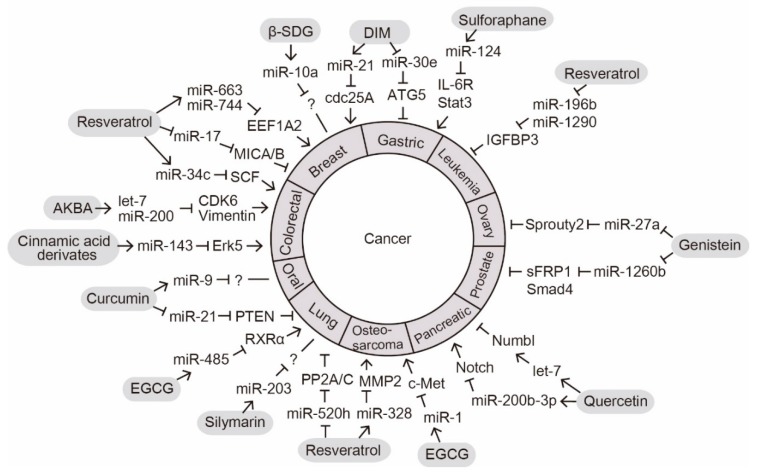
MicroRNA (miRNA)-mediated anticancer functions of phytochemicals. Phytochemicals regulate the expression levels of miRNAs implicated in the development of various cancers. Arrow indicates activation at the levels of expression; lines with a perpendicular line at the end indicates inhibition of expression; question mark indicates an unknown target molecule.

The anticancer effect is a well-known phytochemical function that is important for promoting human health [16]. We summarize the recent reports on phytochemicals that exert their intracellular anticancer effects by altering miRNA expression (Figure 1).

### 3.1. Resveratrol

Resveratrol (*trans*-3, 5, 4′-trihydroxystilbene) is a polyphenol found in various plants, including berries and grapes [17]. Resveratrol is known for its beneficial properties in human health and has been intensively studied for its anticancer function in multiple cancers such as breast, prostate, stomach, pancreas, and thyroid cancers since 1997 [18,19]. Recently, resveratrol was tested in clinical trials for colon cancer [20]. Several studies provide evidence that the anticancer function of resveratrol is mediated by modulating miRNA expression [13,21,22,23,24,25,26].

Yang et al. analyzed changes in the profiles of 754 human miRNAs following resveratrol treatment in osteosarcoma cells [24]. In response to resveratrol, miR-328 is the most highly upregulated miRNA. MiR-328 targets the 3′UTR of matrix metalloproteinase 2 (MMP2), an essential regulator of metastasis. Therefore, resveratrol exerts its anticancer effects, such as inhibition of migration, invasion, and adhesion of osteosarcoma cells, by regulating the expression of miR-328 and MMP2.

In lung cancer cells, miR-520h is downregulated following resveratrol treatment, and consequently its target genes, PP2A/C, are derepressed [25]. PP2A/C inhibits AKT/NF-κB-mediated FOXC2 expression. Since the FOXC2 is a key regulator that activates epithelial-mesenchymal transition (EMT) and metastasis, suppression of FOXC2 by resveratrol-mediated regulation of the miR-520h-PP2A/C axis induces mesenchymal-epithelial transition (MET) and exerts anticancer effects in lung cancer cells.

In the breast cancer cell line MCF7, resveratrol treatment increases the expression levels of miR-663 and miR-744, and inhibits cell proliferation [21]. Vislovukh et al. demonstrated that the putative oncogene, isoform A2 of eukaryotic translation elongation factor 1A (EEF1A2), is a direct target of both miR-663 and miR-744, and downregulation of EEF1A2 negatively affects cell proliferation [21]. Therefore, the anticancer effect of resveratrol on breast cancer progression could be mediated by regulating the expression of miR-663 and miR-744 that suppress EEF1A2. Resveratrol also promotes natural killer (NK) cell-mediated anticancer immune responses in breast cancer [13]. Oncogenic miR-17, which belongs to the miR-17-92 cluster, is transcriptionally upregulated by the binding of c-Myc and promotes proliferation, migration, and angiogenesis of breast cancer cells [13]. Resveratrol downregulates c-Myc expression and consequently miR-17 expression is suppressed. Suppression of miR-17 upregulates its target genes, such as major histocompatibility complex class I chain-related proteins A and B (MICA and MICB). Ultimately, MICA and MICB increase the susceptibility of breast cancer cells to lysis by NK cells.

Resveratrol also affects the proliferation, migration, invasion, and apoptosis of colorectal cancer cells by regulating miR-34c-mediated stem cell factor (SCF) expression [23]. In HT-29 cells in vitro and in mouse xenografts in vivo, exposure to resveratrol increases the level of miR-34c while decreasing the level of SCF, a known target of miR-34c.

It has been reported that the anticancer effect of resveratrol in acute lymphoblastic leukemia (ALL) is mediated by miRNAs [26]. Zhou et al. observed that miR-196b and miR-1290 are highly expressed in T-cell ALL and B-cell ALL, respectively, and their expression is downregulated by resveratrol [26]. Both miR-196b and miR-1290 directly bind to the 3′UTR of insulin-like growth factor binding protein 3 (IGFBP3) and the downregulation of IGFBP3 is associated with the development of leukemia. Therefore, the resveratrol-induced decrease in miR-196b and miR-1290 levels and consequent recovery of IGFBP3 expression might ultimately inhibit the proliferation and migration of ALL cells.

Mitochondrial dynamics are involved in the development of human diseases including cancer; increased fission or decreased fusion leads to the formation of fragmented mitochondria that triggers apoptosis [27]. Resveratrol may exert an anticancer effect by regulating mitochondrial dynamics via modulating miRNA levels [22]. In several cancer cell lines, such as DLD1, HeLa, and MCF-7, resveratrol increases the expression of miR-326, which in turn directly targets a regulator of mitochondrial fusion, pyruvate kinase M2 (PKM2) [28]. In this way, resveratrol promotes cancer cell apoptosis by decreasing mitochondrial fusion induced by the miR-326-PKM2 axis.

### 3.2. (-)-Epigallocatechin-3-Gallate

EGCG is a major polyphenol in green tea and has been shown to have anticancer effects [29]. In a variety of cancer cells, miRNA profiling studies have been performed to determine the role of miRNAs in regulating EGCG-mediated anticancer functions [14,30,31]. In human hepatocellular carcinoma HepG2 cells, 13 miRNAs were upregulated, while 48 miRNAs were downregulated by EGCG [30]. Tsang et al. demonstrated that miR-16 potently promoted apoptosis [30]. EGCG-induced miR-16 expression suppresses the anti-apoptotic protein Bcl-2 level, leading to the apoptosis of HepG2 cells.

Wang et al. found that the expression of miR-210 was prominently increased by EGCG in both human and mouse lung cancer cells in vitro [14]. Upregulation of miR-210 inhibits the proliferation and anchorage-independent growth of lung cancer cells. Zhou et al. further supported the significant role of miRNAs in the anticancer function of EGCG in vivo [31]. EGCG inhibits tobacco carcinogen-induced lung tumors in A/J mice by modulating the expression of 21 miRNAs, including miR-210. Additionally, Jiang et al. showed that EGCG enhances the expression levels of miR-485 which directly targets the retinoid X receptor (RXRα) in non-small cell lung cancer (NSCLC) cells [32]. EGCG-induced miR-485-RXRα axis represses cancer stem cells (CSC)-like characteristics of NSCLC cells, resulting in the inhibition of cell growth and promotion of apoptosis.

The anticancer function of EGCG in suppressing cell proliferation has been shown to be mediated by miRNAs in osteosarcoma and oral squamous cell carcinomas [33,34]. In an miRNA profiling study, miR-1 was identified as a mediator of EGCG anticancer activities [34]. miR-1 levels are downregulated in osteosarcoma tumor tissues and miR-1 is increased by EGCG to target a proto-oncogene c-MET. In oral squamous cell carcinomas, miR-204 is upregulated by EGCG and targets Slug and Sox4, which are associated with cancer stemness and EMT [33].

Moreover, it has been reported that EGCG affects the expression of several miRNAs known to act as oncogenes or tumor suppressors [35,36]. In prostate cancer cells of mice treated with EGCG, oncogenic miR-21 is downregulated and tumor suppressive miR-330 is upregulated [36]. In human malignant neuroblastoma SH-SY5Y and SK-N-DZ cells, oncogenic miR-92, miR-93, and miR-105b are downregulated and tumor suppressive miR-7-1, miR-34a, and miR-99a are upregulated by EGCG [35]. Therefore, EGCG may exert its anticancer effects by regulating miRNAs involved in tumorigenesis.

### 3.3. Curcumin

Curcumin is a natural phytochemical derived from the root and rhizome of *Curcuma longa* and has been known to have antioxidant, anti-inflammatory, and anticancer properties [37,38]. Recent studies suggest that the anticancer effect of curcumin is mediated by modulation of miRNAs. Zhang et al. demonstrated the anti-proliferative and pro-apoptotic activities of curcumin in NSCLC cells via the miR-21-phosphatase and tensin homolog (PTEN) axis [39]. In NSCLC cells, curcumin downregulates the expression of miR-21, which is a typical oncogenic miRNA implicated in cancer development and progression. Downregulation of miR-21 by curcumin results in the derepression of its target gene, PTEN. Since PTEN is a tumor suppressor, the elevation of PTEN expression might mediate the anticancer effects of curcumin [40].

Aberrant activation of the Wnt signaling pathway affects cell growth, the cell cycle, and invasion in carcinogenesis [41]. Curcumin was shown to suppress cancer cell growth by regulating the Wnt signaling pathway [42]. In oral squamous cell carcinoma, miR-9 is downregulated, and curcumin increases miR-9 expression [43]. The upregulation of miR-9 by curcumin elevates the levels of GSK-3β and phosphorylated GSK-3β, resulting in inhibition of the Wnt signaling pathway. Xiao et al. observed that the expression level of cyclin D1, a putative target of miR-9, was reduced when miR-9 was overexpressed in SCC-9 cells, although whether cyclin D1 is targeted by miR-9 via direct binding was not verified [43]. The direct target gene of miR-9 under curcumin-treated conditions remains to be identified.

### 3.4. Quercetin

Quercetin is a flavonoid derived from fruits and vegetables, such as berries, apples, onions, and broccoli [44]. Recently, anticancer functions of quercetin through miRNA modulation were reported in pancreatic cancer cells [45]. Nwaeburu et al. performed a miRNA profiling analysis of pancreatic ductal adenocarcinoma following treatment with quercetin [45]. They found 105 differentially expressed miRNAs in quercetin-treated cells; 80 miRNAs including let-7c, miR-200a-3p, and miR-200b-3p were upregulated, while 25 miRNAs including miR-103a-3p, miR-125b, and miR-1202 were downregulated. Nwaeburu et al. then investigated how let-7c, one of the most highly upregulated miRNAs, inhibits pancreatic cancer progression [45]. While most miRNAs inhibit the expression of their target genes, let-7c directly targets the 3′UTR of Numbl, an inhibitor of Notch expression, and increases the expression of Numbl. Induction of Numbl by let-7c subsequently antagonizes Notch signaling, known to be involved in cell proliferation, angiogenesis, and development [46], thus leading to tumor growth inhibition and increased apoptosis of pancreatic ductal adenocarcinoma cells.

In addition, an anti-proliferative function of miR-200b-3p induced by quercetin in cancer stem cells (CSCs) has been reported [47]. Notch and Numb are crucial for the regulation of CSC self-renewal; Notch is required for symmetric division and Numb is a marker for asymmetric division [48]. Nwaeburu et al. demonstrated that miR-200b-3p targets Notch by directly binding its 3′UTR, which in turn inhibits proliferation and self-renewal of CSCs [47]. Together, these results indicate that quercetin exerts its anticancer effects by regulating Notch signaling via miRNAs.

### 3.5. 3,3′-Diindolylmethane

One of the natural derivatives of indoles from cruciferous vegetables, 3,3′-diindolylmethane (DIM), has been reported to have an anticancer function in various cancer cells [49]. Ye et al. demonstrated that DIM inhibited the proliferation of gastric cancer cells in vitro and tumor growth in vivo in a xenograft mouse model [50]. DIM downregulates miR-30e, which is highly expressed in multiple types of tumors. Autophagy-related gene 5 (ATG5), an essential component for the generation of autophagosomes [51], was validated as a direct target of miR-30e. These observations suggest that the suppression of miR-30e by DIM rescues ATG5 expression and induces autophagy, which in turn inhibits the proliferation of gastric cancer cells. Therefore, autophagy regulation mediated by the miR-30e-ATG5 axis is crucial for the anticancer function of DIM in gastric cancer cells.

In addition, DIM inhibits breast cancer cell growth in vitro and in vivo [52]. DIM treatment inhibits the proliferation of MCF-7 and MDA-MB-468 breast cancer cells, and the growth of transplanted human breast carcinoma cells in a mouse model. The inhibitory effect of DIM on cell proliferation is due to cell cycle arrest. Jin et al. observed that in response to DIM, miR-21 is upregulated and cdc25A, a putative target of miR-21, is downregulated [52]. As cdc25A is a crucial regulator of cell cycle progression, the anticancer function of DIM via cell cycle arrest might be mediated in part by the modulation of miR-21 and cdc25A expression.

### 3.6. Sulforaphane

Sulforaphane (SFN), a dietary phytochemical converted from cruciferous plants, such as broccoli, carrots, and kale, is known to have anticancer functions [53]. Wang et al. reported that SFN enhances the anticancer function of cisplatin, a DNA-targeting cytotoxic platinum compound, by modulating miRNA expression in gastric cancer cells [54]. Treatment with cisplatin alone causes side effects that increase the CSC-like properties of gastric cancer cells by activating the interleukin-6 (IL-6)-mediated signal transducer and activator of transcription 3 (Stat3) signaling; however, cotreatment with SFN improves the chemotherapy efficacy. SFN increases the expression of miR-124, which targets the IL-6 receptor (IL-6R) and Stat3 by directly binding their 3′UTRs. IL-6R is known to mediate activation of the downstream molecules of IL-6 signaling, such as mitogen-activated protein kinase, phosphatidylinositol 3-kinase (PI3K), and Stat3 [55]. Therefore, SFN inhibits the CSC-like viability of gastric cancer cells by regulating the expression levels of miR-124 and its target genes, IL-6R and Stat3.

In breast cancer cells, SFN affects cell cycle arrest, senescence, apoptosis, and autophagy [56]. Lewinska et al. investigated the changes in the miRNA profiles of three breast cancer cell lines, MCF-7, MDA-MB-231, and SK-BR-3, upon treatment with SFN [56]. Sixty miRNAs were upregulated and 32 were downregulated. Specifically, the levels of miR-23b-3p, miR-92b-3p, miR-381-3p, and miR-382 were significantly reduced in the three cell lines. The target genes recognized by these miRNAs that link them to their anticancer functions remain to be identified.

### 3.7. Genistein

Genistein is a major isoflavonoid isolated from soybeans [57]. Genistein has been shown to prevent multiple cancers by regulating the expression of specific oncogenic miRNAs, such as miR-27a and miR-1260b [58,59,60,61,62,63]. Genistein downregulates miR-27a expression in uveal melanoma, ovarian cancer, pancreatic cancer, and lung cancer cells to inhibit cell proliferation, migration, or invasion [60,61,62,63]. Zinc finger and BTB domain (Broad-Complex, Tramtrack and Bric a brac) containing 10 and Sprouty2 were validated as targets of miR-27a in uveal melanoma cells and ovarian cancer cells, respectively [60,62]. Oncogenic miR-1260b is downregulated by genistein in renal and prostate cancer cells [58,59]. The Wnt signaling pathway is typically activated in cancer cells to promote tumorigenesis and miR-1260b targets tumor suppressor genes associated with Wnt signaling, such as *sFRP1*, *Dkk2*, and *Smad4* [58,59]. Therefore, genistein-induced downregulation of miR-1260b rescues the expression levels of target genes that antagonize Wnt signaling and ultimately inhibits cancer cell proliferation and invasion.

### 3.8. Acetyl-11-Keto-β-Boswellic Acid

Boswellic acids, the major components of a gum resin derived from *Boswellia serrata*, have been known to perform anti-inflammatory functions [64]. Takahashi et al. reported that acetyl-11-keto-β-boswellic acid (AKBA), an active component in boswellic acids, has an anticancer function in colorectal cancer cells [64]. AKBA inhibits cell viability, colony formation, proliferation, and migration, and enhances apoptosis in colorectal cancer cells. This anticancer effect is partly mediated by the regulation of well-known tumor suppressive miRNAs, such as the let-7 and miR-200 families [64]. AKBA increases the expression levels of let-7b, let-7i, miR-200b, and miR-200c, and decreases expression of their known target genes implicated in EMT, such as CDK6 and vimentin. Therefore, AKBA-mediated regulation of the let-7 and miR-200 families may play an important role in inhibiting metastasis in colorectal cancer.

### 3.9. Silymarin

Silymarin is a flavonoid isolated from the milk thistle, *Silybum marianum* L. Gaertn, and has been shown to have anticancer activities [65]. Singh et al. reported that silymarin inhibits the migratory activities of NSCLC cells, including A549, H1299, and H460, by modulating the expression of miR-203, a tumor suppressor [65]. Silymarin enhances miR-203 expression and decreases histone deacetylases and zinc finger E-box binding homeobox 1 (ZEB1) expression. As ZEB1 is implicated in the activation of EMT, downregulation of ZEB1 by silymarin might inhibit the metastasis of cancer cells. Although a novel target gene of miR-203 was not identified in the study, it was shown that miR-203 plays its tumor suppressor role in multiple cancers by suppressing its known target genes, such as annexin A4 and apoptosis inhibitor 4 [66,67].

### 3.10. β-Sitosterol-d-glucoside

β-Sitosterol-d-glucoside (β-SDG) is a phytosterol found in *Salvia sahendica* and *Arctotis arctotoides* and reported to possess anti-inflammatory, antimicrobial, immunomodulatory, and anti-proliferative functions in multiple cancer cells [68]. Xu et al. demonstrated that β-SDG isolated from sweet potato has an anticancer function in breast cancer cells [69]. β-SDG inhibits the proliferation of breast cancer cells by elevating the levels of pro-apoptotic Bax and BCL2 associated agonist of cell death (Bad), and reducing the levels of anti-apoptotic Bcl-2 and Bcl-xl. β-SDG also suppresses tumor growth in MCF7 cell-injected xenograft models. Moreover, Xu et al. showed that the expression level of miR-10a is increased by β-SDG, and this induction of miR-10a promotes apoptosis and suppresses PI3K and p-Akt levels [69]. Therefore, the anticancer function of β-SDG is probably mediated in part by miR-10a. A direct target gene of miR-10a involved in its anticancer activities needs to be identified to shed light on the specific molecular mechanism involved.

### 3.11. Arctigenin

Arctigenin is a lignan from the seeds of *Arctium lappa*. Several studies demonstrated the anticancer functions of arctigenin in pancreatic, breast, and lung cancers through the regulation of apoptosis and proliferation [70]. Wang et al. studied the effects of arctigenin on the miRNA profile of mouse prostate tumor tissues; the levels of miR-126 and miR-21 decrease whereas those of miR-135a, miR-205, miR-22-3p, miR-455, and miR-96 increase in response to arctigenin [71]. The molecular mechanism that connects these regulated miRNAs and the anticancer function of arctigenin remains to be elucidated.

### 3.12. Cinnamic Acid Derivatives

Propolis is a plant mastic that contains many chemical components including flavonoids, benzoic acid, and cinnamic acid derivatives such as artepilin C, baccharin, and drupanin [72]. Chemical components isolated from propolis have been shown to possess anti-inflammatory and anticancer properties [72,73]. Kumazaki et al. demonstrated that artepilin C, baccharin, and drupanin inhibit the proliferation of colon cancer cells, such as DLD-1 and SW480 [74]. Specifically, the combined treatment with baccharin and drupanin has a synergistic effect on apoptosis activation in DLD-1 cells. Moreover, the expression level of miR-143 is increased by cotreatment with baccharin and drupanin, and miR-143 subsequently represses the expression of a target gene, Erk5, resulting in cell cycle arrest [74]. These observations support the idea that the pro-apoptotic anticancer function of propolis cinnamic acid derivatives is due to modulation of miRNAs and their target genes.

## 4. Phytochemicals with Photoprotective Effects

In addition to their anticancer effect, phytochemicals have been recently reported to inhibit or protect against UV-induced cellular damage [75,76]. Analysis of the altered miRNA profiles in arctiin- or troxerutin-pretreated keratinocytes upon UVB stimulation suggests that the phytochemical-induced regulation of miRNAs is functionally associated with the photoprotective effect.

### 4.1. Arctiin

Arctiin is a lignan found in plants, such as *Arctium lappa* and *Forsythiae fructus,* and is known to inhibit cell proliferation and inflammation [77,78]. Recently, Cha et al. demonstrated the photoprotective activities of arctiin in keratinocytes, the predominant cell type in the epidermis [75]. Arctiin inhibits cell death and cytotoxicity and enhances DNA repair and wound healing in UVB-exposed keratinocytes. UV radiation induces DNA damage in keratinocytes, which results in cellular senescence, apoptosis, or cancer. MiRNA profile analyses of arctiin-pretreated HaCaT cells under UVB radiation revealed that the expression levels of four miRNAs were increased and those of 62 were decreased (>2-fold) [75]. These results raise the possibility that the protective function of arctiin against UVB-induced cellular damage is mediated by the modulation of miRNA expression.

### 4.2. Troxerutin

Troxerutin {vitamin P4; 3′, 4′, 7′-Tris[O-(2-hydroxyethyl)]}, is a natural flavonoid rutin found in extracts of *Sophora japonica*, which exhibits antioxidant and anti-inflammatory activities [79,80]. Lee et al. demonstrated that troxerutin promotes cell migration and inhibits cell death in UVB-exposed HaCaT cells [76]. Since the expression of miRNAs involved in apoptosis and cell cycle arrest is aberrantly regulated under UVB radiation, Lee et al. hypothesized that the protective function of troxerutin against UVB-induced DNA damage and apoptosis is mediated by modulating miRNA expression [76]. Indeed, miRNA microarray analysis indicated that five miRNAs were upregulated and 63 were downregulated in troxerutin-pretreated HaCaT cells under UVB exposure, suggesting that the photoprotective effect of troxerutin in UVB-induced cellular damage is mediated by miRNA regulation.

## 5. A Phytochemical with Anti-Hepatosteatosis Effects

MiRNAs are known to regulate lipid homeostasis, and the aberrant expression of miRNAs has been examined in multiple metabolic diseases, including obesity and diabetes [81]. Jeon et al. demonstrated that fisetin (3, 3′, 4′, 7-tetrahydroxyflavone), a natural flavonol found in various fruits and vegetables, regulates lipid metabolism by modulating miRNA expression [82]. In high-fat mouse liver, the levels of five miRNAs: miR-22*, miR-146a, miR-146b, miR-802, and miR-378, are increased, and fisetin suppresses the expression of miR-378 [82]. In addition, Jeon et al. identified nuclear respiratory factor-1 (NRF-1), a transcription factor implicated in mitochondrial function, as a target of miR-378 [82]. These results suggest that a high-fat diet increases miR-378 levels and the consequent repression of NRF-1 expression leads to mitochondrial dysfunction and development of hepatosteatosis (hepatic fat accumulation). Furthermore, Jeon et al. provide evidence that this metabolic alteration is rescued by fisetin via modulation of miRNA expression [82].

## 6. Discussion and Conclusions

**Figure 2:**
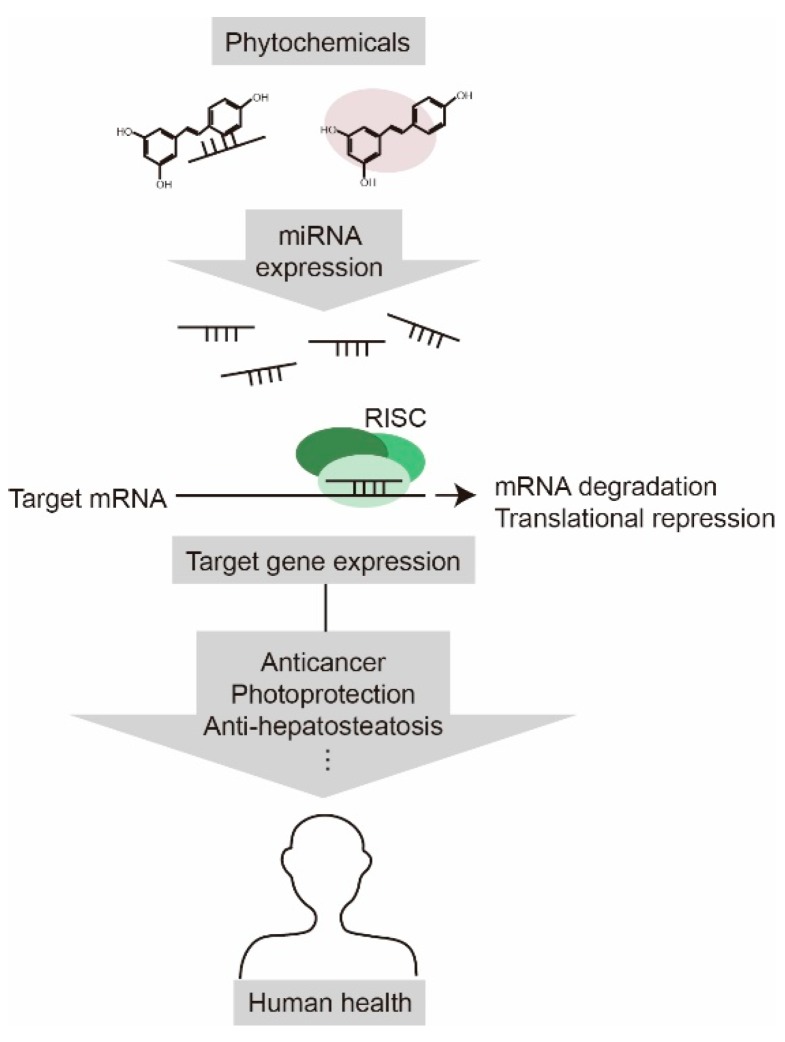
Phytochemicals have a positive impact on human health by regulating miRNA expression. Phytochemicals alter the gene expression profile by modulating miRNA expression, which cause anticancer, photoprotection, and anti-hepatosteatosis effects.

We provided an overview of the recent research on miRNA-mediated health-promoting effects of phytochemicals (Figure 2). There have been efforts to elucidate the molecular mechanisms underlying the regulation of miRNA expression by phytochemicals. To date, the majority of studies have been focused on the phytochemical-induced changes in miRNA profiles in pathological conditions such as cancer, and more profiling data continue to emerge. Since miRNAs play an important role in maintaining normal cell physiology as well as inducing pathological conditions [83,84], it is perhaps not surprising that phytochemicals exert their medicinal effects through miRNA regulation. As a single miRNA can affect numerous target mRNAs, and a phytochemical can play a wide range of regulatory roles via miRNAs in cells. Elucidating the molecular mechanisms underlying the health-promoting effect of phytochemicals by identifying the crucial regulatory miRNAs and their targets will facilitate the efforts to maximize the therapeutic benefits of phytochemicals.

Understanding the anticancer function of phytochemicals mediated by miRNA regulation is currently an active area of research. As several miRNAs function as either oncogenic miRNAs or tumor suppressors [11], phytochemicals could exert their anticancer effects by directly controlling miRNA expression. In general, miR-21, miR-17, miR-30e, and miR-520h function as oncogenic miRNAs, whereas miR-200, miR-34c, miR-143, and let-7 function as tumor suppressors [11]. One phytochemical could regulate several oncogenic or tumor suppressive miRNAs in various cancers. For example, resveratrol controls miR-520h, miR-17, miR-328, miR-196b, miR-1290, miR-34c, miR-663, and miR-744 in leukemia, osteosarcoma, lung, prostate, breast, and colorectal cancers [13,21,23,24,25,26]. On the other hand, a typical oncogenic or tumor suppressive miRNA may be modulated by multiple phytochemicals, as in the case of miR-200, whose expression is regulated by at least two phytochemicals, AKBA and quercetin [64].

The phytochemical SFN enhances the anticancer function of the conventional therapeutic drug cisplatin by modulating miRNAs [54]. Moreover, combined treatment with two phytochemicals, baccharin and drupanin, exerts a synergistic anticancer effect in colorectal cancer cells [74]. These results suggest that phytochemicals possess great potential for development as effective health-promoting and anticancer therapeutic agents. However, since the majority of studies have focused on identifying miRNAs modulated by phytochemicals, future studies are required to explore the direct targets of the miRNAs and verify their associated functions. For example, although SFN, silymarin, β-SDG, and arctigenin exert anticancer effects, and arctiin and troxerutin exert a photoprotective effect through controlling miRNA expression, the direct targets of the miRNAs have yet to be thoroughly investigated [54,65,69,71,75,76].

Finally, little is known about the mechanism by which the expression levels of miRNAs are modulated by phytochemicals. A few studies suggested that phytochemicals can bind to miRNAs directly or affect the transcription or processing of miRNAs by regulating a third molecule [12,13,14]. Interestingly, genistein regulates specific microRNAs, such as miR-27a and miR-1260b, in various cancer cells to elicit anticancer functions [58,59,60,61,62,63]. A distinct mechanism likely exists to regulate specific microRNA expression. If so, understanding the molecular mechanism by which genistein regulates the expression of specific miRNAs will be useful for controlling multiple types of cancers. Therefore, more extensive studies of the molecular mechanisms responsible for regulating miRNA expression levels are warranted.

## Abbreviations

miRNAs	microRNAs
Bax	BCL2 associated X
UTR	untranslated region
MMP2	matrix metalloproteinase 2
EMT	epithelial-mesenchymal transition
MET	mesenchymal-epithelial transition
EEF1A2	isoform A2 of eukaryotic translation elongation factor 1A
NK	natural killer
MICA	major histocompatibility complex class I chain-related protein A
MICB	major histocompatibility complex class I chain-related protein B
ALL	acute lymphoblastic leukemia
IGFBP3	insulin-like growth factor binding protein 3
PKM2	pyruvate kinase M2
NSCLC	non-small cell lung cancer
PTEN	phosphatase and tensin homolog
DIM	3,3′-diindolylmethane
CSC	cancer stem cell
ATG5	autophagy-related gene 5
SFN	sulforaphane
Stat3	signal transducer and activator of transcription 3
IL-6R	interleukin-6 receptor
PI3K	phosphatidylinositol 3-kinase
AKBA	acetyl-11-keto-β-boswellic acid
β-SDG	β-Sitosterol-d-glucoside
NRF-1	nuclear respiratory factor-1
